# Publisher Correction: Negative differential resistance as a critical indicator for the discharge capacity of lithium-oxygen batteries

**DOI:** 10.1038/s41467-019-08964-x

**Published:** 2019-02-22

**Authors:** Yoko Hase, Yasuhiro Komori, Takayoshi Kusumoto, Takashi Harada, Juntaro Seki, Tohru Shiga, Kazuhide Kamiya, Shuji Nakanishi

**Affiliations:** 1 Toyota Central R&D Labs., Inc., 41-1 Yokomichi, Nagakute, Aichi 480-1192 Japan; 2 Research Center for Solar Energy Chemistry, Osaka University, 1-3 Machikaneyama, Toyonaka, Osaka 560-8531 Japan; 3 Department of Chemistry, Graduate School of Engineering Science, Osaka University, 1-3 Machikaneyama, Toyonaka, Osaka 560-8531 Japan

Correction to: *Nature Communications* 10.1038/s41467-019-08536-z, published online: 05 February 2019

The original version of this Article contained an error in the title, which was previously incorrectly given as “Negative differential resistance as a critical indicator for the discharge capacity of lithium-oxygene batteries”. The correct version states “lithium-oxygen” in place of “lithium-oxygene”. This has been corrected in both the PDF and HTML versions of the Article.

