# Supplementary material for: Negative differential resistance as a critical indicator for the discharge capacity of lithium-oxygen batteries
Source: Nat Commun. 2019 Feb 5;10:596. doi: 10.1038/s41467-019-08536-z (PMC6363801; doi:10.1038/s41467-019-08536-z)
Supplement: Supplementary file 1 — Supplementary Information [file 41467_2019_8536_MOESM1_ESM.pdf]

Supplementary Information for

Negative differential resistance as a critical indicator for the discharge capacity of lithium-oxygen batteries

Yoko Hase et al.

Supplementary Figures

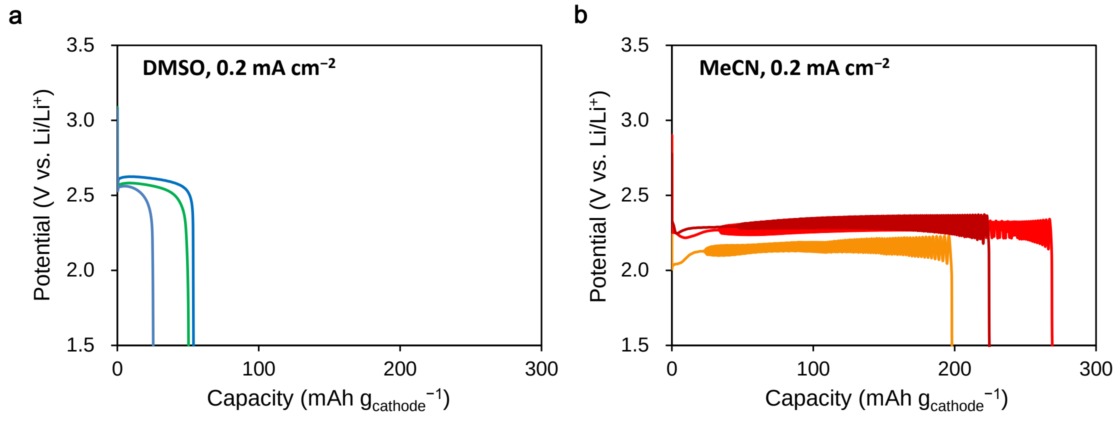

Supplementary Figure 1 | Discharge curves obtained in MeCN/CP and DMSO/CP systems. Discharge curves of DMSO (**a**) and MeCN (**b**) systems with 0.5 M of LiTFSI. The applied current density was 0.2 mA cm^{-2}. The measurements were performed for 3 times for each condition.

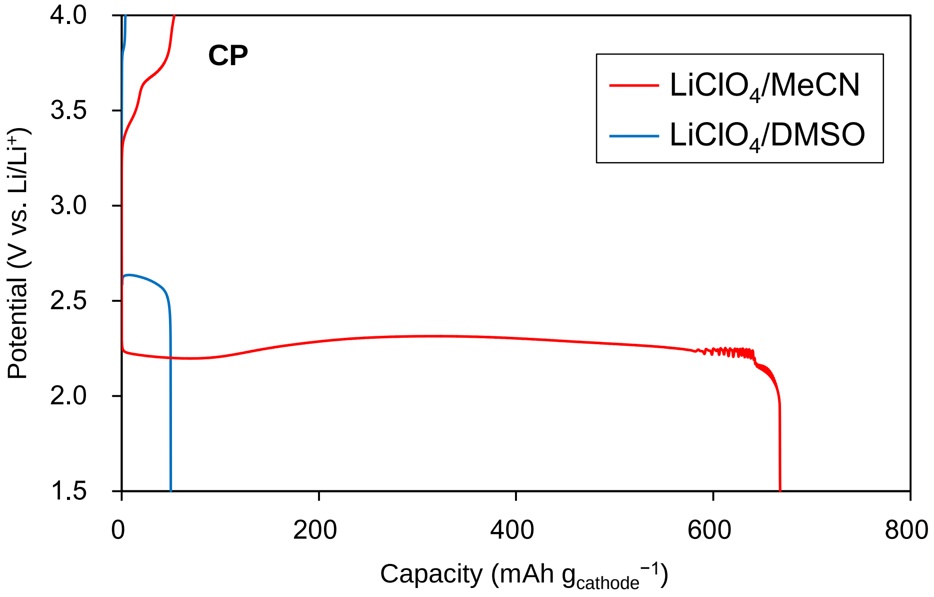

Supplementary Figure 2 | Discharge curves obtained in MeCN/CP and DMSO/CP systems. Galvanostatic discharging was performed with CP in MeCN or DMSO with 0.5 M of $LiClO_4$. The applied current density was 0.2 mA cm^{-2}.

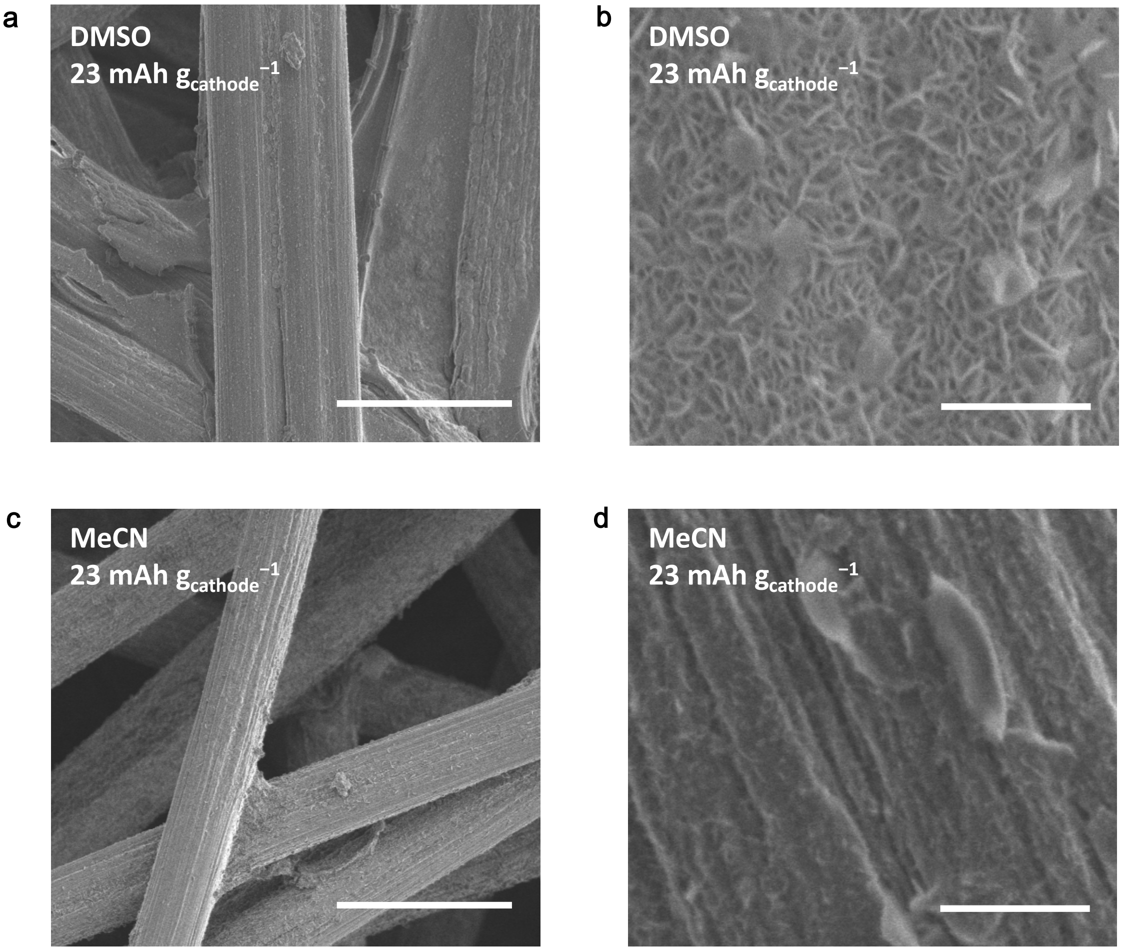

Supplementary Figure 3 | Cathode substrate effects on morphology of discharge product. SEM images of CP obtained after discharge. The bare basement of CP was remained in **c** and **d**. In contrast, the whole surface was covered with Li_2O_2 deposition in **a** and **b**. The scale bars, 20 μm (**a, c**) and 0.5 μm (**b, d**).

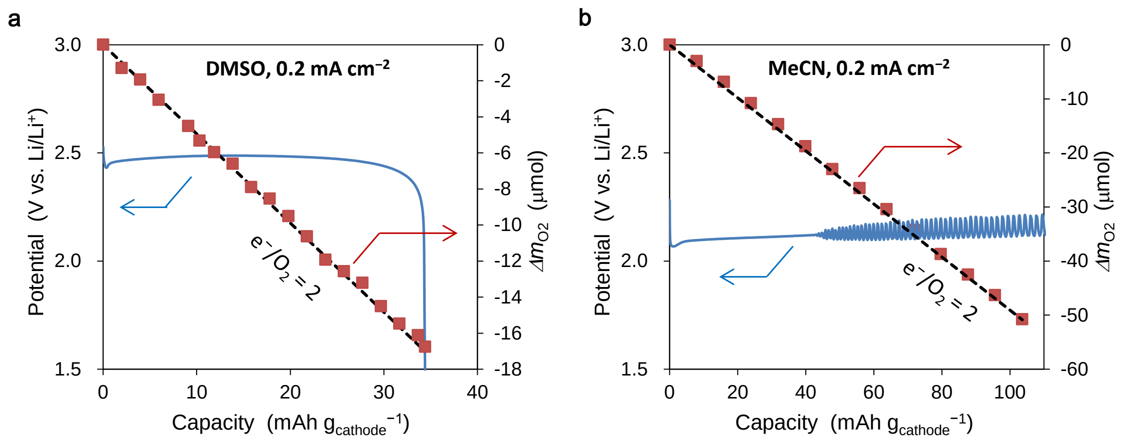

Supplementary Figure 4 | Galvanostatic discharge curves and O_2 consumption. Blue lines are galvanostatic discharge curves obtained with CP cathodes in 0.5 M LiTFSI/DMSO (**a**) and 0.5 M LiTFSI/MeCN (**b**). Red squares are plots of O_2 consumption measured using pressure decay during discharge. The dashed lines indicates theoretical value of O_2 consumption when the discharge reaction was proceed with $2e^{-}/O_2$ reduction process.

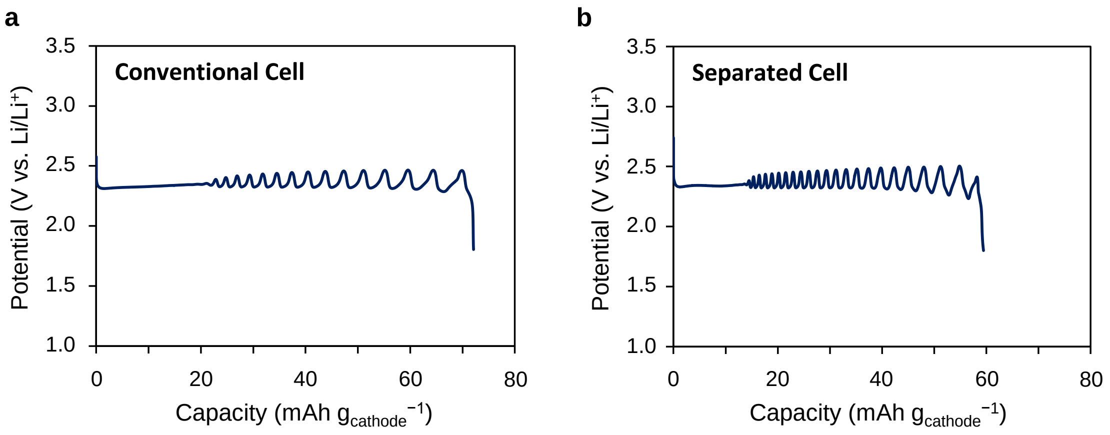

Supplementary Figure 5 | Discharge curves of Li-O_2 cells with CP cathode in superconcentrated electrolyte (LiTFSI/MeCN, molar ratio 1/2) at a discharge rate of 0.01 mA cm^{-2}. The discharge capacities were obtained from galvanostatic discharging to a reductive potential of 1.8 V using a conventional cell with polyethylene film separator (a) and a separated cell with LIC-GC separator (b). The oscillation behavior was observed in the results using both type of cells, therefore, the specific discharge behavior in LiTFSI/MeCN electrolyte was independent of cell structures and LIC-GC.

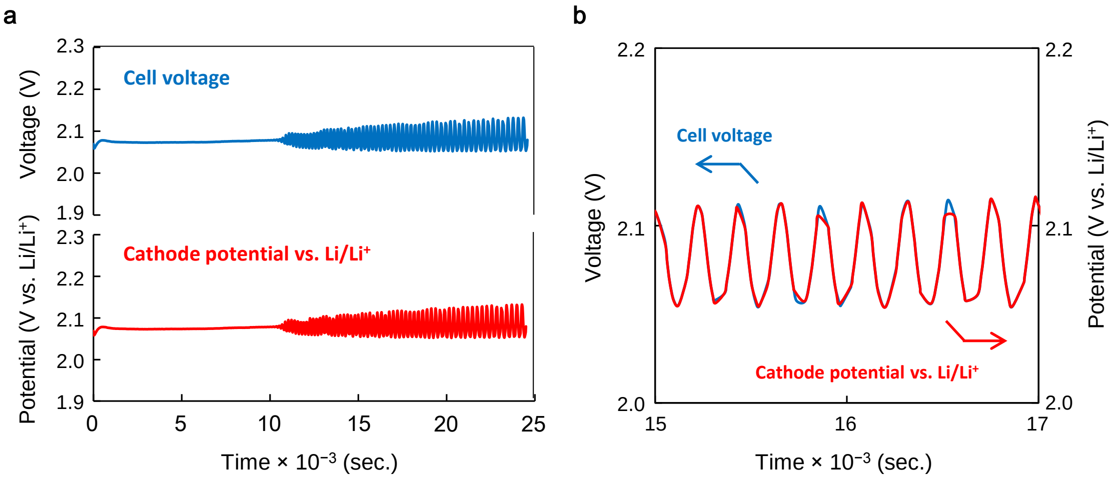

Supplementary Figure 6 | Discharge curves of Li-O_2 cells with CP cathode in 0.5 M LiTFSI/MeCN at a discharge rate of 0.2 mA cm^{-2}. a, Cell voltage (blue) and cathode potential versus Li/Li^+ (red) during galvanostatic discharging were plotted with time. **b,** The oscillation curves of the cell voltage (blue) and the cathode potential of the cell (red). The oscillation behavior of the cell voltage was consisted with the cathode potential.

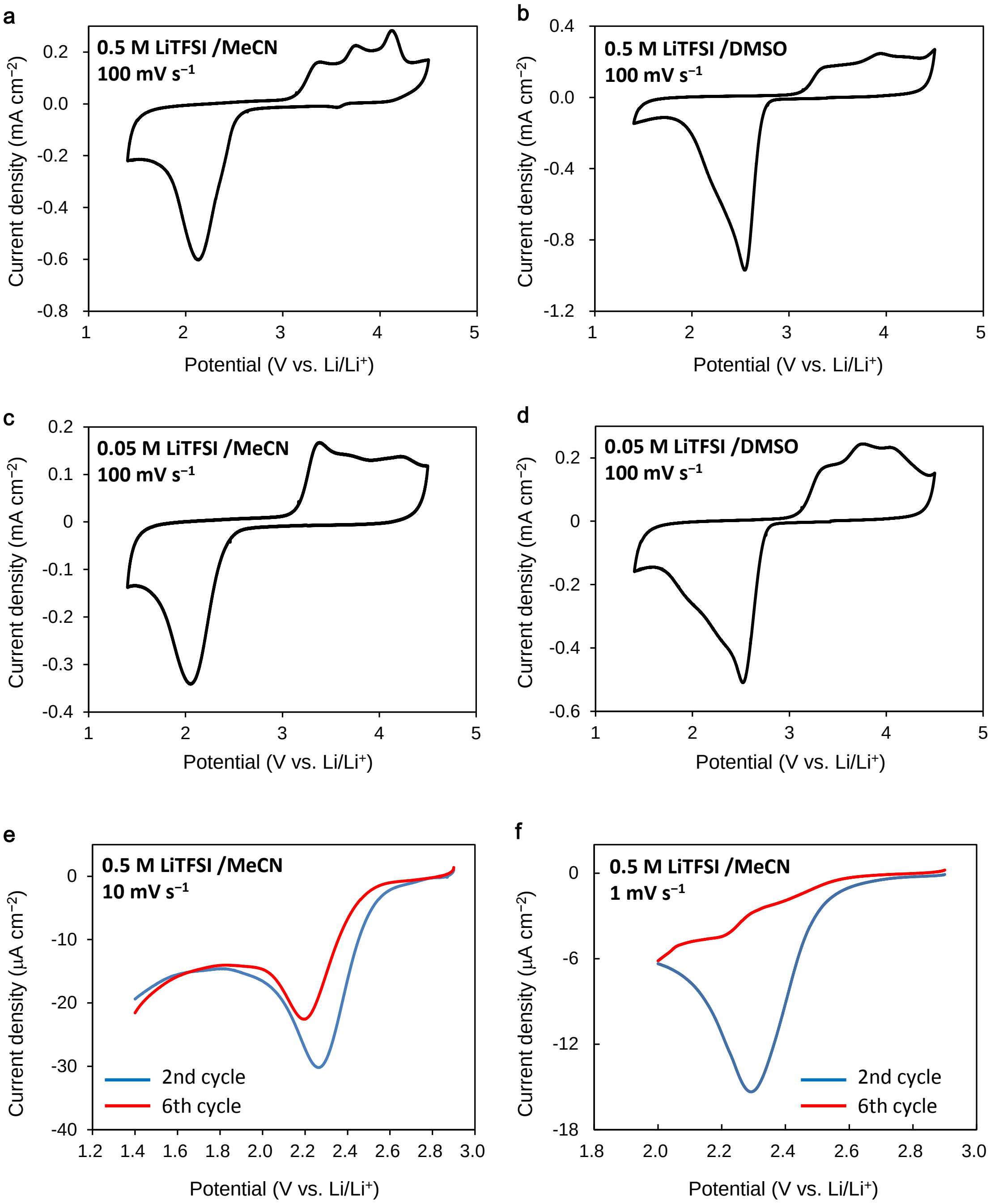

Supplementary Figure 7 | CVs measured with Glassy carbon electrode obtained in the LiTFSI/MeCN electrolyte. CVs for 2nd cycle measured with glassy carbon working electrode in 0.5 M LiTFSI/MeCN (**a**), 0.5 M LiTFSI/DMSO (**b**), 0.05 M LiTFSI/MeCN (**c**), and 0.05 M LiTFSI/DMSO (**d**). The scan rate was 100 mV s^{-1}. (**e, f**) CV (the negative sweep) for 2nd and 6th cycles measured with glassy carbon working electrode in 0.5 M LiTFSI/MeCN. The applied scan rates were 10 mV s^{-1} (**e**) and 1 mV s^{-1} (**f**).

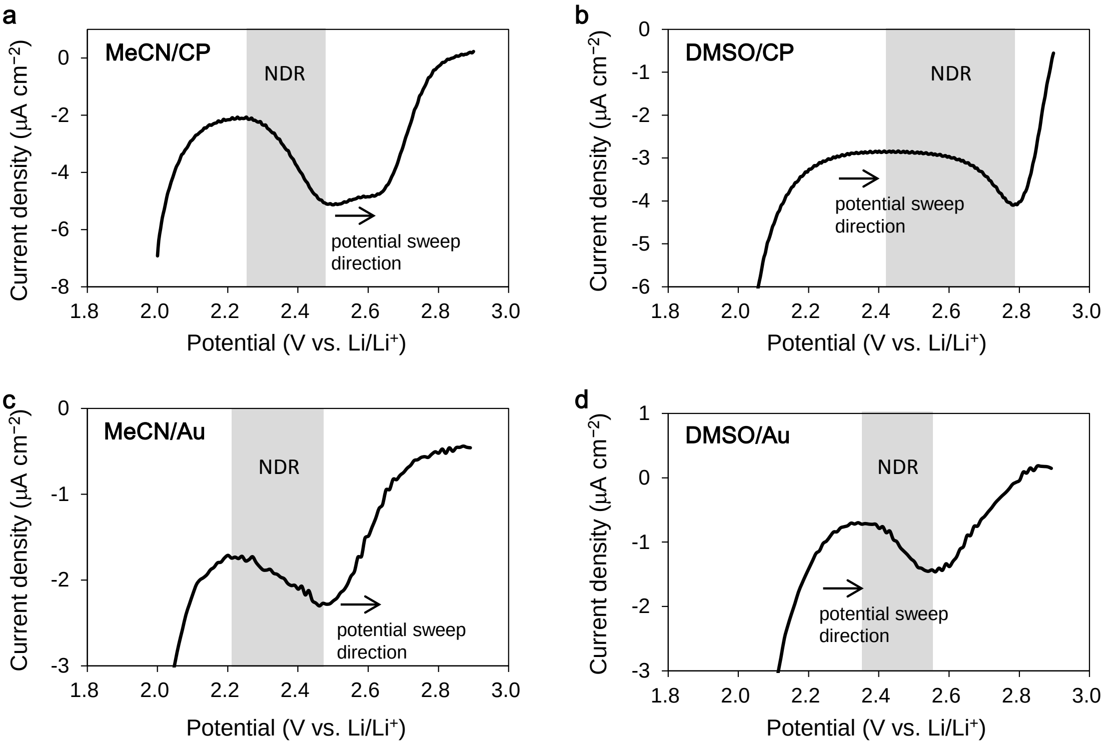

Supplementary Figure 8 | Voltammograms in the NDR potential region. Voltammograms (the positive sweep) for MeCN/CP (**a**, 18th cycle), DMSO/CP (**b**, 14th cycle), MeCN/Au (**c**, 1st cycle), and DMSO/Au (**d**, 1st cycle) systems. The scan rate was 0.5 mV s^{-1}.

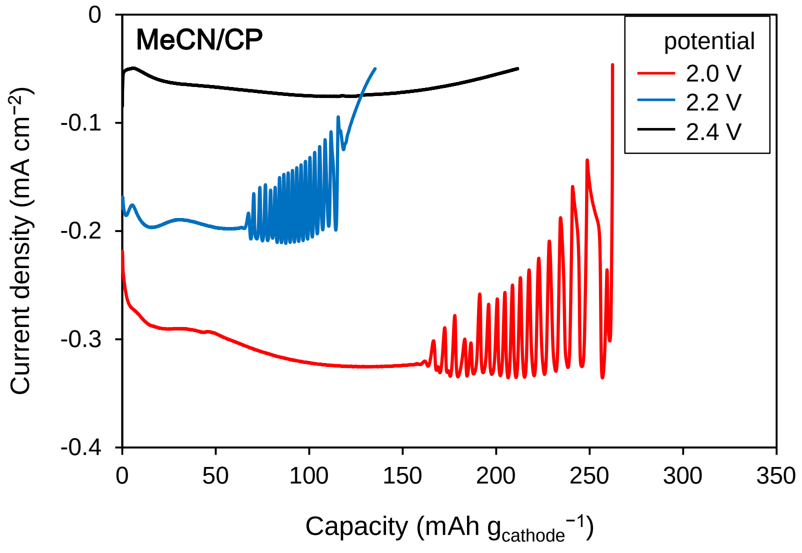

Supplementary Figure 9 | Potentiostatic discharging at various potentials. Potentiostatic discharge curves were obtained with CP cathode in 0.5 M LiTFSI/MeCN electrolyte.

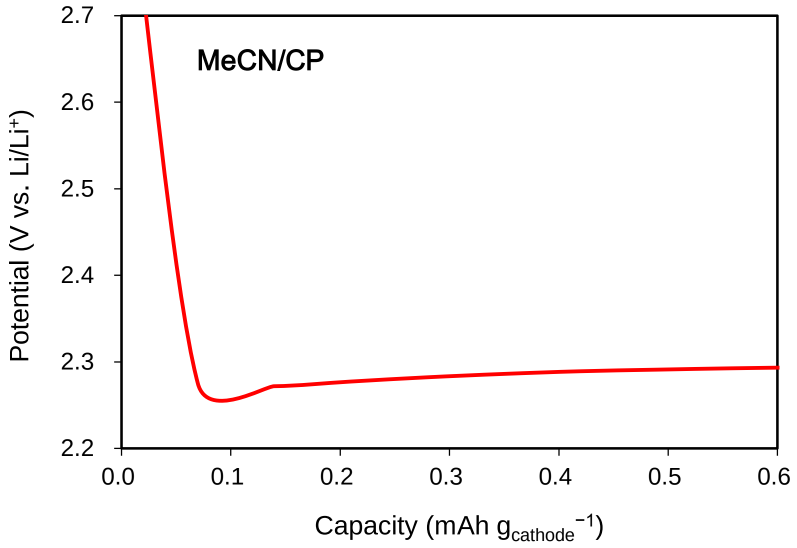

Supplementary Figure 10 | Initial stage of galvanostatic discharge curves. Galvanostatic discharge curves obtained with CP cathode in 0.05 M LiTFSI/MeCN. The applied current densities were 0.2 mA cm^{-2}.

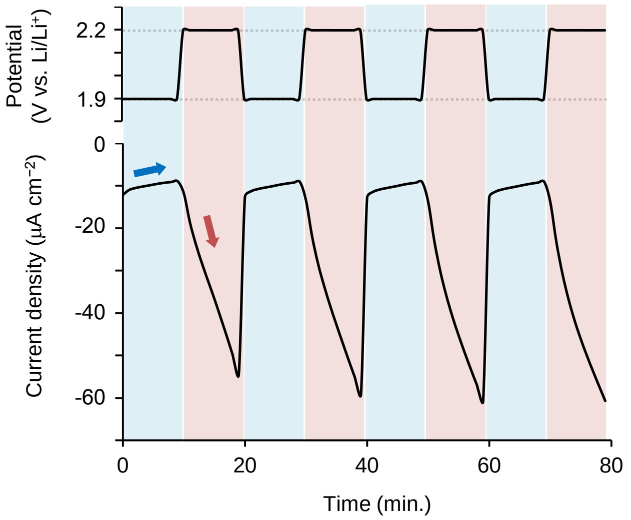

Supplementary Figure 11 | The time course of the ORR current for the MeCN/CP system. The potentials of the potentiostatic discharging were switched to 1.9 or 2.2 V at 10-minute intervals. The measurement was performed after potentiostatic discharging at 1.9 V (the potential region of surface pathway, see Fig. 5) with pre-discharging treatment.

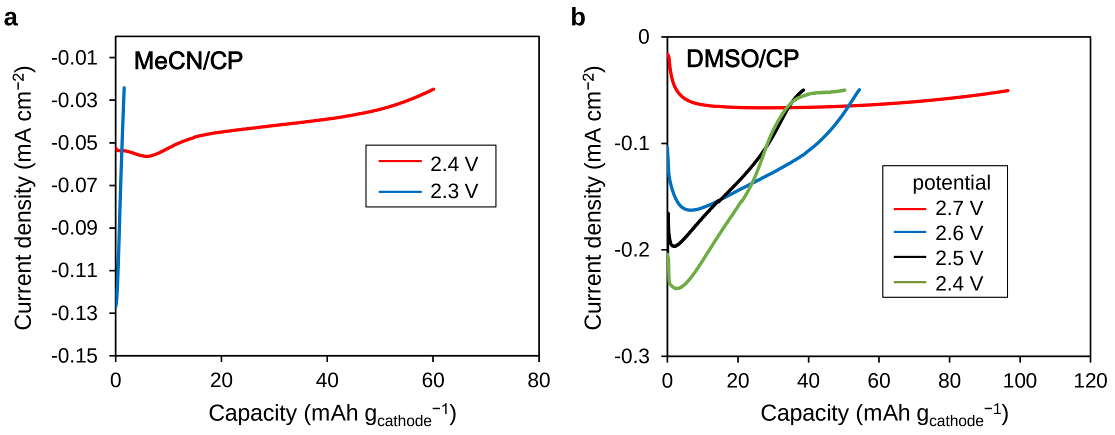

Supplementary Figure 12 | Potentiostatic discharging without pre-discharging in 0.05 M LiTFSI/electrolyte. a, The potentiostatic discharge capacities were precipitously changed between 2.3 V and 2.4 V before pre-discharging because NDR potential region placed there in MeCN electrolyte, whereas it placed between 1.9 V and 2.0 V after pre-discharging as shown in Fig. 5b. The obtained current densities was relatively smaller than Fig. 5b. **b,** The same trend of discharge behavior was obtained before (Fig. 5c) and after pre-discharging in DMSO electrolyte.

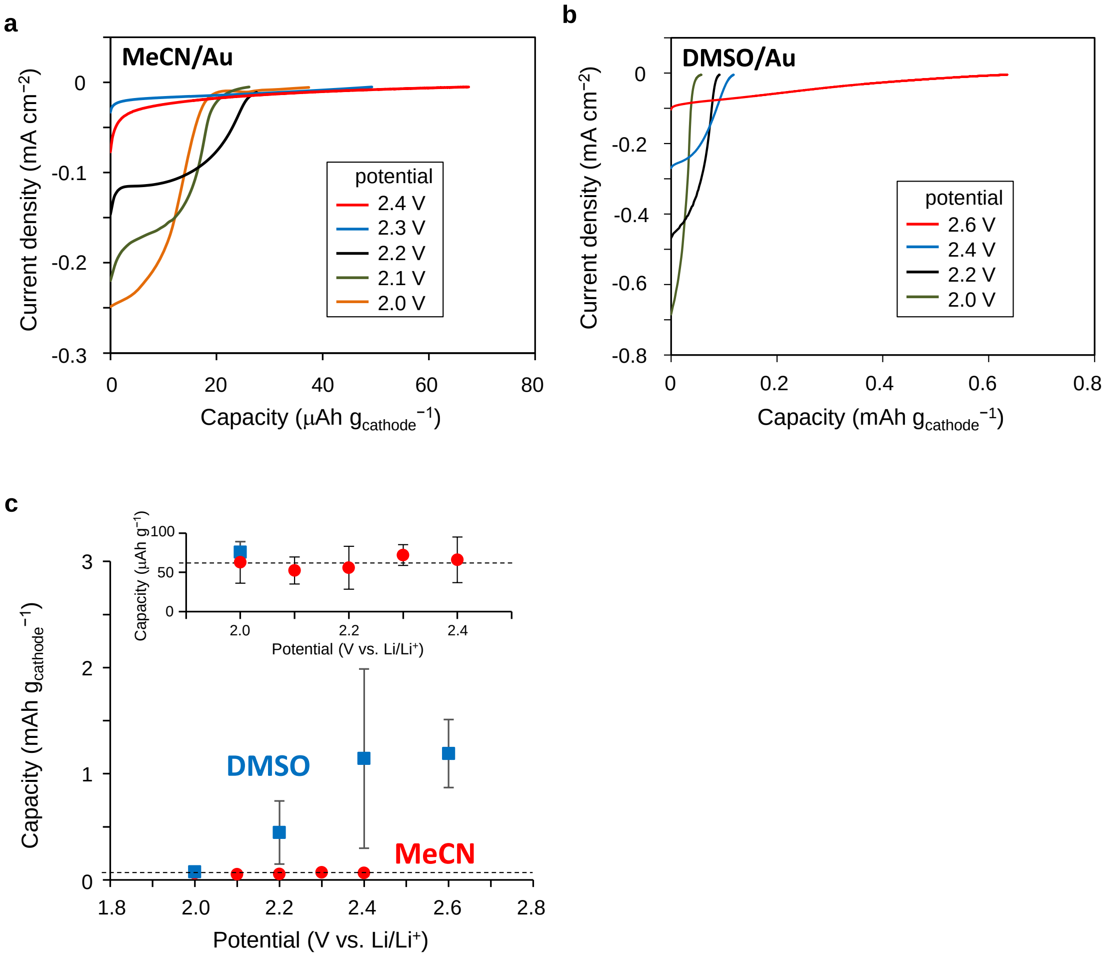

Supplementary Figure 13 | Potentiostatic discharging behavior of Au cathode systems. (**a, b**) Potentiostatic discharge curves obtained in 0.5 M LiTFSI electrolytes without pre-discharging. The cut off current density was 0.005 mA cm^{-2}. **c**, Discharge capacities of potentiostatic discharging at various potentials corresponding to Supplementary Fig. 13a (MeCN, red circles) and 13b (DMSO, blue squares). The inset shows the magnified image of **c**. The dashed lines indicate the theoretical capacity via the surface pathway (61 μAh $g_{cathode}^{-1}$).

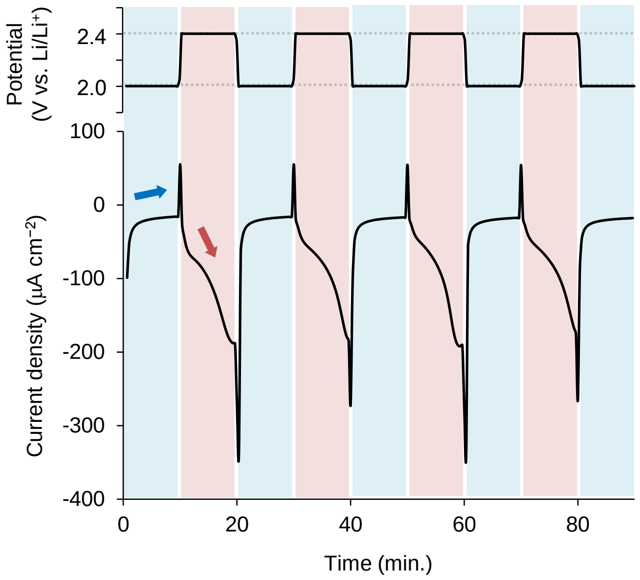

Supplementary Figure 14 | The time course of the ORR current for the MeCN/CP system shown in Fig. 4b. The current spikes originated from capacitive effect with potential switching were not removed from the raw data.

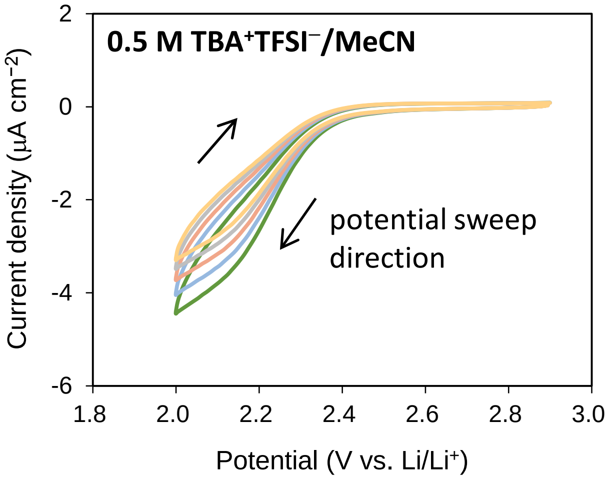

Supplementary Figure 15 | The CVs obtained under the same condition of Fig. 4a without Li^+. The CVs of 5th to 10th cycles were measured in the electrolyte of 0.5 M tetrabutylammonium TFSI salt in MeCN (0.5M TBA^+TFSI^-/MeCN) using 2-electrode set up with CP for working electrode. The potential scan rate was 0.5 mV s^{-1}. Li^+ gradually diffused from the anode side during the measurement, however, the effect can be ignored in this measurement period.

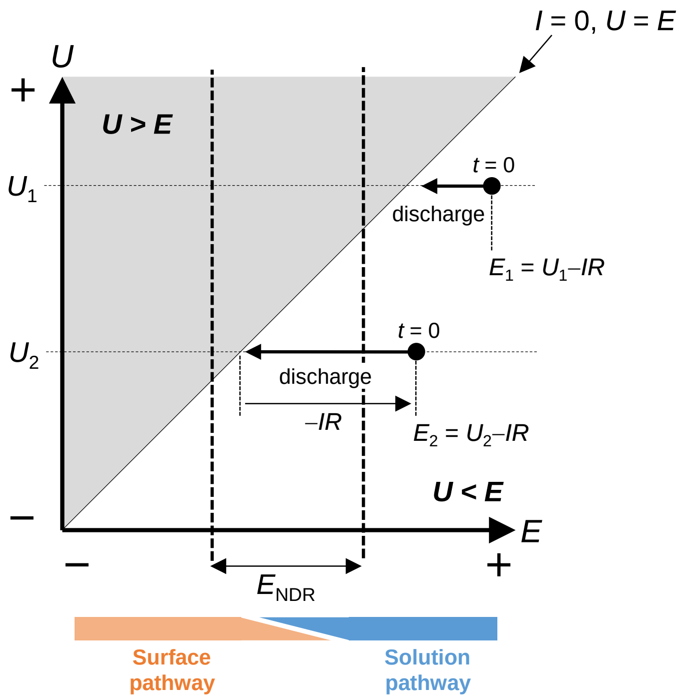

Supplementary Fig. 16 | Schematic diagram representing the relationship between *U* and *E* on potentiostatic discharging. The grey and white colored areas shows the potential region of $U > E$ and $U < E$, respectively. In the potentiostatic discharging of Li-O_2 batteries, the *U* value is constant while the current *j* is negative, therefore, *E* is always placed in the potential region of $U < E$.

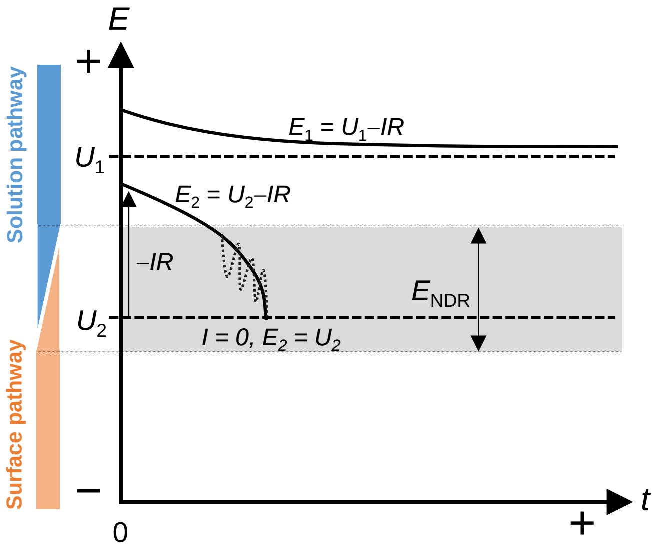

Supplementary Figure 17 | Schematic diagram representing the time course of *E* on potentiostatic discharging. The grey colored area shows the potential region of NDR (E_{NDR}). *U* is set on constant value and *E* is un-observable and un-controllable while *E* determines the ratio of reaction pathways.

Supplementary Table 1: Discharge Li_2O_2 yields (Y_{Li2O2}) for cells employing CP cathode.

electrolyte	Y_{Li2O2}(%)[*]
0.5 M LiTFSI/MeCN	81.5 ± 0.8
0.5 M LiTFSI/DMSO	80.6 ± 1.1

[*] The data in Table S1 were calculated using samples obtained by discharging in the range of 0.7 mAh to 1.3 mAh. The values were reported with standard error of at least three replicate trials. Li_2O_2 yields (Y_{Li2O2}) are calculated using the following equation:

$$Y_{Li2O2} = n_{Li2O2,titration}/n_{Li2O2,e}$$

$n_{Li2O2,e}$ is the amount of Li_2O_2 expected to form via a $2e^-/Li_2O_2$ process during discharge.

Supplementary Notes

Views on the Possible Effects of Contamination with Water. It is well known that a trace amount of water in an electrolyte can result in an increased discharge capacity via the activation of the solution route to the formation of Li_2O_2 [1]. Because water can potentially be found in battery systems, it is important to confirm that the high capacity observed when using the low-DN MeCN electrolyte in the present work was not due to the effect of any water that may have been present. As described in the main text, Fig. 5e summarizes the dependence of the discharge capacity on the positive electrode potential. Importantly, the discharge capacity was significantly different within the NDR potential region, even though all conditions other than the electrode potential were held constant. In particular, at potentials more negative than the NDR potential region, the experimental discharge capacity equaled the theoretical value predicted in the case that the reaction proceeds solely via the surface pathway. The effect of contamination by water can also be evaluated by cyclic voltammetry (CV). If water is present in a battery system, the CV data obtained at 0.05 mV s^{-1} will contain an additional peak at approximately 2.3 V[1]. However, this peak was not observed in our experimental work (Supplementary Fig. 7). The above results clearly indicate that water was not present in the battery system in the present research to the extent that contamination by water would change the main conclusion.

Mechanism of the Potential and Current Oscillation. Oscillation phenomena in electrochemical systems associated with NDR is a research field that has been studied for a long time as summarized in some reviews[2-7]. It has been mathematically clarified that the existence of NDR is a required condition for the potential and current oscillation to be occurred[8-13]. It should be noted here that the current and potential oscillations can appear in an electrochemical system with NDR, irrespective of the molecular origin of the NDR.

The mechanism of the potential oscillation can be explained within the framework of the general theory of electrochemical oscillation. According to the general theory of electrochemical oscillation[10], NDR and the second factor which hides the NDR on *I-U* curves are the two of necessary conditions for the appearance of spontaneous potential oscillation. This type of electrochemical oscillation is categorized as “hidden-NDR type” in the literatures[5,7,10]. In this case, the inhibiting effect of LiO_2* is the origin of NDR and the second factor is Faraday current which is attributed to the decomposition of the solvent[5]. In accordance with the general theory, the

mechanism of the appearance of NDR in this manuscript is described as below. (In this theoretical explanation, the decrease of the active electrode area due to the accumulation of Li_2O_2 during discharge is ignored.) During galvanostatic experiments, the current is constant (i.e., the ohmic drop, *IR*, is constant, where *R* is the resistance of the solution between the electrode surface and the reference electrode and *I* is negative for the reduction current). Therefore, the true electrode potential (or Helmholtz double layer potential), *E*, changes in the same manner as the positive electrode potential, *U* (since $U = E + IR$). At higher values of *U* (and also of *E*), the inhibiting effect is low, as coverage by the LiO_2* is lower, and therefore the oxygen reduction reaction (ORR) proceeds efficiently. However, a high ORR rate will decrease the surface concentration of oxygen and thus causes a gradual negative shift in the potential so as to maintain a constant current density. In the NDR region, this negative potential shift leads to increases in coverage by the LiO_2* (i.e., the inhibitor) and so decreases the ORR current. This, in turn, results in a further negative shift of the potential, in a positive-feedback manner, to maintain a constant current. Since the total current is regulated to be constant under a galvanostatic experiment, the contribution of the current derived from the solvent decomposition to the regulated current value increases with the precipitous negative shift of *E* corresponding to the inhibiting effect of LiO_2* on ORR. Thus, the potential rapidly changes to the negative end of the potential oscillation due to the positive feedback. Since ORR is suppressed at the potential which is close to the negative end of potential oscillation, the surface concentration of O_2 at the electrode gradually increases. Such increase of the concentration of O_2 on the effective electrode surface leads to the increase of the ORR current contribution to the total current values, therefore, the potential turns to the positive direction over time. The rapid change of the potential from the negative- to positive-end of the potential oscillation can be explained in exactly the same way. In the NDR region, this positive potential shift leads to decrease in coverage by the LiO_2* (i.e., the inhibitor) and so increase the ORR current and decrease of the current derived from the decomposition of the solvent. This, in turn, results in a further positive shift of the potential, in a positive-feedback manner, to maintain a constant current.

The mechanism responsible for the current oscillation (Supplementary Fig. 9) can also be explained in the similar framework. According to the general theory of electrochemical oscillation[10], the second factor, which hides NDR, is unnecessary. The appearance of current oscillation at a constant applied potential requires an ohmic drop (*IR*) in the electrolyte[5]. It should first be noted that a constant positive electrode potential, *U*, was maintained via an external potentiostat in the present

work. During the high (absolute value) current stage of the oscillation, *E* was much more positive than *U* due to the ohmic drop (since *U* = *E* + *IR*, where *I* is negative for the reduction current). The active ORR during the high-current stage decreased the surface concentration of dissolved oxygen, leading to a gradual decrease in the absolute value of *I* and thus to a decrease in the ohmic drop and a negative shift in *E*. This negative shift in *E* increased coverage by the LiO_2*. Because LiO_2* can act as an inhibitor for the ORR (as described in the main text), the value of *I* was further decreased. Thus, *I* decreased in an autocatalytic fashion as a result of the positive feedback manner. During the low-current stage, the ORR proceeds only slowly at vacant sites; thus, the surface concentration of dissolved oxygen gradually increases via the diffusion of oxygen from the bulk solution. This increase in the surface concentration induced an increase in *I* that, in turn, caused a positive shift in *E*. The positive shift in *E* led to the desorption of LiO_2*, resulting in a further increase in *I*. Based on this positive feedback mechanism, the value of *I* increased rapidly, after which the high-current stage appeared again.

It should be noted that this positive-feedback process can proceed most efficiently if the NDR value is high. NDR is not a sufficient but a required condition for electrochemical oscillation. A positive feedback mechanism that destabilizes the system is necessary for the appearance of electrochemical oscillation phenomena. Importantly, the larger NDR appears, the more effective positive feedback mechanism works, eventually inducing electrochemical oscillation. The relationship between the existence of the NDR and the appearance of oscillation phenomenon was not only shown by experimental results but also proved by mathematical theory[8-10]. In the present work, both potential and current oscillations were observed in the MeCN/CP system, which exhibited a high NDR (Fig. 5d).

The Characteristic Potentiostatic Discharge Profile Associated with the NDR Potential Region. As noted above, there was a difference between the observable and controllable potential, *U*, and the unobservable and uncontrollable potential, *E*. This represents an important factor in the reaction, due to the ohmic drop, *IR* (since *U* = *E* + *IR*). Essentially, *U* and *E* have the same value only in the case that there is no current flow (*I* = 0). During the cathodic reaction, meaning the discharge reaction, the value of *E* is always more positive than the *U* value because *I* is negative during discharge. As shown in Fig. 5, during the discharge reaction of Li-O_2 batteries in conjunction with a regulated potential, the observed current decreases with time. That is, *E* (the dominant factor in the reaction) is shifted

toward more negative values with decreasing current.

Supplementary Figs. 16 and 17 explain these phenomena using schematics. Considering a scenario in which the initial set potential, U_1, is greater than E_{NDR}, the current decreases as the discharge progresses, such that E gradually approaches U_1. It should be noted that E never enters the E_{NDR} region in the case that $U_1 > E_{NDR.}$ Therefore, the solution pathway is continuously dominant. The red and blue lines in Fig. 5b, the red and blue lines in Fig. 5c, and the red lines in Supplementary Figs. 12a and b correspond to this case. In contrast, if U_2 is within the E_{NDR} or at a more negative potential, a different profile appears. The solution pathway is dominant immediately after the onset of discharge, at which point the E values are more positive than the E_{NDR} region. However, E is shifted toward more negative values as the current decreases with discharge, such that E eventually enters the E_{NDR} region. Once this occurs, the positive feedback loop described in the above section begins, and there is a precipitous decrease in the current. This series of events corresponds to the black and green lines in Fig. 5b, the black and green lines in Fig. 5c, and the blue line in Supplementary Fig. 12a, where a steep decrease in the current is clearly evident. This positive feedback is a necessary condition for the appearance of current oscillation. In fact, current oscillation was frequently observed in conjunction with a steep decrease in the current under regulated potential conditions (Supplementary Fig. 9). Therefore, the relationship between the value of U and the E_{NDR} region can be determined by examining the time course of the current profile, particularly the presence or absence of a steep decrease in current.

An important aspect of this work is the observation that the discharge continues above a specific potential while the current decreases abruptly below the same potential (sometimes accompanied by current oscillation) under regulated potential conditions. That is, the NDR is present within the potential region over which the transition between the solution and surface reaction pathways certainly occurs.

Possible Effect of Pre-deposition on Potentiostatic Discharging. Potentiostatic experiments shown in Fig. 5a were carried out using a reduced Li salt concentration of 0.05 M because using the same Li salt concentration as in Fig. 1 (i.e., 0.5 M), would frequently generate the spontaneous current oscillations seen in Supplementary Fig. 9, such that the discharge capacity at each potential could not be evaluated correctly. Even in electrolytes containing 0.05 M LiTFSI, the discharge capacity was confirmed to be larger in the MeCN/CP system than in the DMSO/CP system (Fig. 5a).

Rinaldi et al. reported that the Li_2O_2 growth history significantly affects the discharge capacity[14]. More specifically, in the case that an electrode is pre-covered by seeds slowly grown at a lower over-potential, the discharge reaction can be sustained over a longer time span. This result indicates that the system history can greatly modify the subsequent discharge characteristics.

During the potentiostatic discharge trials, we examined the effect of the initial state of the CP surface. In the MeCN/CP system data in Fig. 5a, the positive electrode potential changes from positive to negative during the initial stage of galvanostatic discharge (~0.1 mAh $g_{cathode}^{-1}$), and thereafter stabilizes at a constant potential (Supplementary Fig. 10). Considering these results, it was anticipated that the potentiostatic discharge could be characterized under the same conditions as applied during galvanostatic discharge, based on the following discharge process. Firstly, the cell was pre-discharged at a constant potential in the potential region corresponding to the initial stage of the galvanostatic discharge (at 2.4 V), so as to form seeds on the bare CP substrate. After generating these seeds via discharging at 1 mAh $g_{cathode}^{-1}$ at 2.4 V, the operating potential was changed to various values.

Supplementary Fig. 12 shows the potential dependence of the discharge capacity obtained when a naked CP substrate was used. Here, the discharge capacities were significantly smaller and the NDR potential region located at more negative potentials compared with the results obtained using a pre-covered electrode (as in Figs. 5b and d). These differences can be attributed to the larger over-potential required for the formation of seeds on naked CP substrates[15]. In contrast, this history effect was not observed in the DMSO system (Fig. 5c). As shown in Fig. 5d, since the reaction proceeds through the solution pathway over the entire potential region in the DMSO system, pre-discharge has a minimal effect on the successive discharge (Supplementary Fig. 12), unlike the behavior observed in the MeCN system.

Supplementary Notes for Supplementary Figs. 5 and 6. The potential oscillation shown in Fig. 1b and Supplementary Figs. 1 and 4 were also observed even in a one-compartment cell without the Li-ion conductive membrane, in which super-concentrated MeCN electrolyte ([MeCN]/[LiTFSI] = 2) was used for improving the reductive stability (Supplementary Fig. 5)[16]. The cathode potential against the Li-reference electrode measured using a three-electrode system, which indicates that the oscillations of the cell voltage and the cathode potential were perfectly identical, is shown in Supplementary Fig. 6. These results indicate that the potential oscillation reflected the behavior of the cathode reaction.

Supplementary Methods

Materials. All materials were used as purchased without further purification and stored under argon (Ar). Ketjenblack (ECP600JD) and PTFE binder (F104) were obtained from Mitsubishi Chemical (Tokyo, Japan) and Daikin Industries (Osaka, Japan), respectively. Sheet type cathodes (Ketjenblack sheet cathodes) were prepared by mixing uniformly Ketjenblack (90 wt%) and PTFE binder (10 wt%) in a mortar, and stretched with a thickness of 80 μm and a diameter of 18 mm. Separator film (E25MMS, 25 μm of thickness) was purchased from Tonen Chemical (Tokyo, Japan). Anhydrous dimethyl sulfoxide (DMSO) (water content < 0.001%) was obtained from Wako (Osaka, Japan). *N*,*N*-Diethyl-*N*-methyl-(2-methoxyethyl)ammonium bis(trifluoromethanesulfonyl)imide (DEME-TFSI) (water content < 0.02%) was purchased from Kanto Chemical (Tokyo, Japan). Tetraethylene glycol dimethyl ether (TEGDME, lithium battery grade) was obtained from Kishida Chemical Co., Ltd. (Osaka, Japan).

Electrochemical Measurements. For cyclic voltammetry with a standard 3-electrode setup, the measurements were performed in accordance with previous report[17]. Typical procedure was described below. 3 mm diameter glassy carbon discs (BAS Inc.) were employed as the working electrodes. Prior to use, the working electrodes were polished with 0.05 mm alumina slurry in ethanol and dried in vacuo for 10 min after rinse with ethanol. A platinum mesh served as the counter electrode. Measurements were performed using a reference electrode based on $LiFePO_4$ which was pre-oxidized (50% of total capacity) to Li_xFePO_4 before use. The pre-oxidized Li_xFePO_4 reference was confirmed to show a constant potential at 3.45 V vs. Li/Li^+. The cell was assembled in an Ar glovebox and the electrolyte saturated with O_2 was introduced, and then sealed in a hermetically sealed glass container to maintain dry Ar atmosphere during measurements. Electrochemical measurements were carried out in the sealed glass container set in atmospheric condition at room temperature.

Chemical Titration. The chemical titration processes of Li_2O_2 were performed in accordance with previous report[18,19]. For the titration of Li_2O_2 formed on cathode, the cells were disassembled in an Ar glovebox and the discharged cathodes and electrolytes were extracted from the cells. The volatile materials involving in a sample were evaporated in vacuo without washing of the sample. The sample was put into a glass tube sealed with septa rubber lid and taken out of the glovebox. 2.0 ml of ultrapure deionized water was injected using a syringe under Ar atmosphere and the solution was

stirred for 30s. The involved reaction is as follows:

$Li_2O_2 + 2H_2O \rightarrow 2LiOH + H_2O_2$

The whole titration was processed in the following two steps: (1) acid-base titration and (2) iodometric titration. For the acid-base titration, the base was titrated using a standardized 5 mM HCl solution, with the end point indicated by 0.1 mL of phenolphthalein in ethanol. The involved reaction is as follows:

$LiOH + HCl \rightarrow LiCl + H_2O$

For the iodometric titration, three reagents were added into the solution in sequence: 1 mL of 2 wt% KI aqueous solution, 1 mL of 3.5 M H_2SO_4 solution and 50 μL of Mo-based catalyst solution. The Mo-based catalyst solution was prepared by dissolving 0.5 g ammonium molybdate ($(NH_4)_2MoO_4$) and 1.5 g of ammonium nitrate (NH_4NO_3) into 5 mL of 6N ammonia aqueous solution, then diluting the solution to 25 mL total using ultrapure deionized water. 0.5 mL of 1% starch solution was added to the tubes and the quantity of I_2 formed in the sample solution was titrated by 10mM $Na_2S_2O_3$ aqueous solution. The involved reaction is as follows:

$H_2O_2 + 2KI + H_2SO_4 \rightarrow I_2 + K_2SO_4 + 2H_2O$

$I_2 + 2Na_2S_2O_3 \rightarrow Na_2S_4O_6 + 2NaI$

The results obtained by these titration experiments were summarized in Supplementary Table 1.

Estimation of O_2 Gas Consumption. The amount of O_2 consumed during discharge can be calculated by the ideal gas law using the pressure decay measurement combined with an accurately calibrated headspace volume of the cells. The values of gas pressure in the cells were obtained with a pressure sensor (KEYENCE, AP-44) attached to the cells.

Supplementary References

1. Aetukuri, N.B. et al. Solvating additives drive solution-mediated electrochemistry and enhance toroid growth in non-aqueous Li–O_2 batteries. *Nat. Chem.* **7**, 50−56 (2015).
2. Hudson, J.L. & Tsotsis, T.T. Electrochemical reaction dynamics: a review. *Chemical Engineering Science* **49**, 1493−1572 (1994).
3. Koper, M.T.M. *Advances in Chemical Physics, I* (John Wiley & Sons, Inc., 1996).
4. Krischer, K. *Modern Aspects of Electrochemistry* (Kluwer Academic / Plenum Publishers, 1999).
5. Krischer, K. *Advances in Electrochemical Sciences and Engineering* (Wiley-VCH, 2003).
6. Kiss, I.Z., Nagy, T. and Gáspár, V. *Solid State Electrochemistry II* (Wiley-VCH Verlag GmbH & Co. KGaA, 2011).
7. Orlik, M. *Self-Organization in Electrochemical Systems I* (Springer-Verlag Berlin Heidelberg, 2012).
8. Koper, M.T.M. Stability study and categorization of electrochemical oscillators by impedance spectroscopy1. *J. Electroanal. Chem.* **409**, 175−182 (1996).
9. Koper, M.T.M. Non-linear phenomena in electrochemical systems. *J. Chem. Soc., Faraday Trans.* **94**, 1369−1378 (1998).
10. Strasser, P., Lübke, M., Raspel, F., Eiswirth, M. & Ertl, G. Oscillatory instabilities during formic acid oxidation on Pt(100), Pt(110) and Pt(111) under potentiostatic control. I. Experimental. *J. Chem. Phys.* **107**, 979−990 (1998).
11. Strasser, P., Eiswirth, M. & Koper, M.T.M. Mechanistic classification of electrochemical oscillators — an operational experimental strategy. *J. Electroanal. Chem.* **478**, 50−66 (1999).
12. Mukouyama, Y., Nakanishi, S., Chiba, T., Murakoshi, K. & Nakato, Y. Mechanisms of Two Electrochemical Oscillations of Different Types, Observed for H_2O_2 Reduction on a Pt Electrode in the Presence of a Small Amount of Halide Ions. *J. Phys. Chem. B* **105**, 7246−7253 (2001).
13. Mukouyama, Y., Nakanishi, S., Konishi, H., Ikeshima, Y. & Nakato, Y. New-Type Electrochemical Oscillation Caused by Electrode-Surface Inhomogeneity and Electrical Coupling as Well as Solution Stirring through Electrochemical Gas Evolution Reaction. *J. Phys. Chem. B* **105**, 10905−10911 (2001).
14. Rinaldi, A., Wijaya, O., Hoster, H. & Yu, D.Y.W. History effects in Li-O_2 batteries – how initial seeding influences the discharge capacity. *Chem. Sus. Chem.* **7**, 1283−1288 (2014).

15. Gallant, B.M. et al. Influence of Li_2O_2 morphology on oxygen reduction and evolution kinetics in Li–O_2 batteries. *Energy Environ. Sci.* **6**, 2518−2528 (2013).
16. Yamada, Y. et al. Unusual Stability of Acetonitrile-Based Superconcentrated Electrolytes for Fast-Charging Lithium-Ion Batteries. *J. Am. Chem. Soc.* **136**, 5039−5046 (2014).
17. Johnson, L. et al. The role of LiO_2 solubility in O_2 reduction in aprotic solvents and its consequences for Li–O_2 batteries. *Nat. Chem.* **6**, 1091−1099 (2014).
18. McCloskey, B.D. et al. Combining Accurate O_2 and Li_2O_2 Assays to Separate Discharge and Charge Stability Limitations in Nonaqueous Li−O_2 Batteries. *J. Phys. Chem. Lett.* **4**, 2989−2993 (2013).
19. Qiao, Y. et al. From O_2^- to HO_2^-: Reducing By-Products and Overpotential in Li-O_2 Batteries by Water Addition. *Angew. Chem., Int. Ed.* **56**, 4960−4964 (2017).
